# Lower Posttraumatic α-Synuclein Level Associated With Altered Default Mode Network Connectivity Following Acute Mild Traumatic Brain Injury

**DOI:** 10.3389/fncir.2019.00026

**Published:** 2019-04-16

**Authors:** Limei Ye, Danbin Zhang, Meihua Shao, Pinghui Zhao, Bo Yin, Jinfei Zhuang, Feifei Wang, Zhihan Yan, Guanghui Bai

**Affiliations:** ^1^Department of Radiology, The Second Affiliated Hospital and Yuying Children’s Hospital of Wenzhou Medical University, Wenzhou, China; ^2^Department of Radiology, Jinhua Municipal Central Hospital and Jinhua Hospital of Zhejiang University, Jinhua, China; ^3^Department of Radiology, Tongde Hospital of Zhejiang Province, Hangzhou, China; ^4^Department of Neurosurgery, The Second Affiliated Hospital and Yuying Children’s Hospital of Wenzhou Medical University, Wenzhou, China; ^5^Department of Rehabilitation, The Second Affiliated Hospital and Yuying Children’s Hospital of Wenzhou Medical University, Wenzhou, China; ^6^Department of MRI, The First Affiliated Hospital of Zhengzhou University, Zhengzhou, China

**Keywords:** mild traumatic brain injury, α-synuclein, rs-fMRI, default mode network, functional connectivity

## Abstract

This study aimed to investigate the changes of α-synuclein in serum and its relationship with default mode network (DMN) connectivity after acute mild traumatic brain injury (mild TBI). Fifty-two patients with mild TBI at the acute phase and 47 matched healthy controls were enrolled in the study. All participants received resting-state functional magnetic resonance imaging (fMRI) and neuropsychological assessments. Relations between the levels of α-synuclein in serum and clinical assessments were obtained using multivariate linear regression. Results showed that the patients with lower α-synuclein presented more complaints on post-concussion symptoms and depression. Moreover, patients with high levels of α-synuclein exhibited significantly decreased functional connectivity in the left precuneus and increased functional connectivity in both the left anterior cingulate cortex and ventro-medial prefrontal cortex (MPFC) compared with patients with low levels of α-synuclein. These findings supported that α-synuclein may modulate the functional connectivity within the DMN and suggest the feasibility of using α-synuclein as an objective biomarker for diagnosis and prognosis of mild TBI.

## Introduction

Traumatic brain injury (TBI) is a major public health problem affecting approximately 1.6 million people every year in the United States. Most (about 80%) are diagnosed with mild TBI (mTBI; Langlois et al., 2006), among whom 10%–20% suffer from persistent headaches, difficulty of thinking, memory problems, attention deficits, mood swings, and frustration (McAllister, 2008; Zemek et al., 2013). However, mTBI diagnosis can be missed by conventional computed tomography and magnetic resonance imaging (Lee et al., 2008). Consequently, it is challenging to predict who will suffer from persistent symptoms after mTBI.

mTBI was recently found to probably cause progressive neurocognitive dysfunction (Mac Donald et al., 2017), leading to an increased risk of developing neurodegenerative and psychiatric diseases (Gardner and Yaffe, 2015), including Parkinson’s disease (PD). However, the pathophysiological mechanism underlying mTBI and its relationship with the risk of developing PD remain unclear. An evaluation of some 1,900 studies determined the plausibility of developing PD under moderate to severe TBI (Institute of Medicine, 2009). However, only “suggestive/limited evidence” was found relating mTBI with clinical diagnosis of PD (Institute of Medicine, 2009). Other studies have investigated the risk of developing PD from mTBI but obtained inconsistent findings (Gardner et al., 2015). Magnoni and Brody (2010) suggested that these results may partly be derived from the putative linkage between TBI and neurodegeneration, with the occurrence of TBI increasing the risk of neurodegenerative diseases.

Several biomarkers are available to diagnose and assess TBI, including imaging and fluid-based techniques (Kochanek et al., 2008). Supporting studies unveil some signatures of these biomarkers associated with TBI. Among these biomarkers, α-synuclein (α-syn) is the hallmark of a number of neurodegenerative diseases (Tokuda et al., 2010; Mollenhauer et al., 2011). Being a presynaptic protein activated by phosphorylation and other pathways, α-syn plays an important role in the circulation of synaptic vesicles. Cellular and genetic modification eventually leads to an overexpression of the total α-syn, and its accumulation has been proven to cause neuronal damages (Werner and Engelhard, 2007; Klein and Westenberger, 2012). In particular, the overexpression of α-syn may be a pathological link between TBI and the development of PD pathologies (Acosta et al., 2015). These results provide a reliable basis for us to investigate mTBI through the study of serum α-syn.

Resting-state functional magnetic resonance imaging (fMRI) has enabled the evaluation of brain networks without task-based fMRI experiments. The default mode network (DMN) is a well-recognized brain network activated during the resting states and suppressed during the execution of attention and decision-making tasks (Zhang and Raichle, 2010). The DMN typically comprises the posterior cingulate cortex (PCC), precuneus, inferior parietal, and medial prefrontal cortex (MPFC) areas (Ralchle and Snyder, 2007). Many studies have determined that several psychiatric disorders alter DMN functional connectivity (Buckner et al., 2008; Rocca et al., 2010; Slobounov et al., 2011). In addition, Zhou et al. (2012) verified significantly reduced connectivity in the PCC and parietal regions, and increased frontal connectivity around MPFC in the mTBI patients (Zhou et al., 2012). Likewise, Mayer et al. (2011) found decreased connectivity within the DMN and increased connectivity between its nodes and the lateral PFC in the mTBI patients (Mayer et al., 2011). However, few studies have addressed the mechanism of changes in the DMN functional connectivity in the mTBI patients. Despite being challenging, this type of study would be insightful for characterizing mTBI. Our main hypothesis was that serum α-syn concentration in the mTBI patients during acute phase will change, and that this inflammatory marker is correlated with clinical neurocognition and functional connectivity within the DMN in the brain.

## Materials and Methods

### Participants

Acute head trauma patients from the local emergency department (ED) in August 2016 and June 2017, with non-enhanced head CT, consecutively became the initial patients in the present study. Inclusion criteria for the mTBI patients were based on guidelines from the World Health Organization’s Collaborating Centre for Neurotrauma Task Force (Holm et al., 2005): (i) an initial Glasgow Coma Scale (GCS) score of 13–15; (ii) one or more of the following: loss of consciousness (LOC) <30 min, post-traumatic amnesia (PTA) <24 h, and/or other transient neurological abnormalities such as focal signs and seizure; and (iii) within 1 week after onset of mTBI. Exclusion criteria for mTBI patients included: (i) pre-TBI, pre-existing psychological disorders, Posttraumatic stress disorder (PTSD), substance abuse, and alcohol dependance; and (ii) a structural abnormality on neuroimaging (CT and MRI); (iii) manifestation of mTBI as complication from other injuries (e.g., systemic injuries, facial injuries, or spinal cord injury); and (iv) other problems (e.g., psychological trauma, language barrier, or coexisting medical conditions), or caused by penetrating craniocerebral injury. Overall, 52 patients with mTBI were enrolled in this study.

Healthy controls were recruited by the local imaging research facilities. Forty-seven age- and gender-matched healthy control participants without neurologic impairment or psychiatric disorders were enrolled.

All participants were right-handed according to the Edinburgh Handedness Inventory (Espírito-Santo et al., 2017). All subjects gave written, informed consent in person approved by the Research Ethics Committee of Second Affiliated Hospital of Wenzhou Medical University and conducted in accordance with the Declaration of Helsinki.

### Serum α-Synuclein Collection

Serum samples for patients and controls were collected in the morning. All participants also did not take any medications. Samples were aliquoted and stored at −80°C until the time of assay after collection and centrifugation. The levels (pg/mL) of α-syn in serum were measured using reagents on a Luminex multiplex bead system (Luminex Corporation, Austin, TX, USA). Serum α-syn assay was performed at Covance using a commercially available ELISA Assay Kit (Covance, Dedham, MA, USA; Mollenhauer et al., 2013). A fluorescence detection laser optic system was used to analyze the binding of each individual protein on the microsphere simultaneously, which permits multiplexed analysis of each of the analytes in one sample. Immunoassays were performed according to manufacturer’s protocols. Intra- and inter-assay coefficients of variation observed for Luminex quantification were less than 20 percent and 25 percent, respectively. Samples with levels that were undetectable by the assay were set to 0.0001 pg/mL. The levels of α-syn >3 standard deviations above and below the population mean within group were considered outliers and excluded for all analysis (Diamond et al., 2015).

### Neuropsychological Tests

For all participants, comprehensive cognitive tests were performed within 48 h of blood sample collection and MRI acquisition. These tests included: (i) Trail-Making Test Part A and Digit Symbol Coding (DSC) score from the Wechsler Adult Intelligence Scale-III (WAIS-III), to examine cognitive information processing speed; (ii) Forward Digit Span (FDS) and Backward Digit Span from the WAIS-III, to assess working memory (Harman-Smith et al., 2013); (iii) Verbal Fluency Test, to assess verbal fluency including language ability, semantic memory, and executive function (Joy et al., 2004); (iv) Beck Depression Inventory-II (BDI-II), to assess depression severity (Reiland, 2017); (v) PTSD Checklist—Civilian Version (PCL-C; Weathers et al., 1991); and (vi) Fatigue Severity Scale (Krupp et al., 1989) and Insomnia Severity Index (ISI; Sadeghniiat-Haghighi et al., 2014). In addition, post-concussive symptoms (PCS) were measured with the Rivermead Post-Concussion Symptom Questionnaire (RPQ; King et al., 1995).

### Image Acquisition

Following acute head injury, non-contrast CT scans were performed on all consecutive patients with a 64-row CT scanner (GE, Lightspeed VCT). MRI scans were acquired using a 3.0T MRI scanner (GE 750). A custom-built head holder was used to prevent head movements. All participants were instructed to remain in a relaxed state, to avoid engaging in any mental activities, and to keep their eyes closed. Alertness during the scan was confirmed immediately after the whole scan was completed. MRI protocols involved the high-resolution T1-weighted 3D MPRAGE sequence (echo time (TE) = 3.17 ms, repetition time (TR) = 8.15 ms, flip angle = 9°, slice thickness = 1 mm, field of view (FOV) = 256 mm × 256 mm, matrix size = 256 × 256), single-shot, gradient-recalled echo planar imaging (EPI) sequence with 54 slices covering the whole brain (TR = 2,000 ms, TE = 30 ms, slice thickness = 3 mm, flip angle = 90°, FOV = 216 mm × 216 mm, matrix size = 64 × 64, voxel size = 3 mm × 3 mm × 3 mm), and axial FLAIR (TR = 9,000 ms, TE = 95 ms, flip angle = 150°, thickness = 5 mm, slices = 20, FOV = 240 mm × 240 mm, matrix size = 173 × 256).

The presence of focal lesions and cerebral microbleeds was determined by an experienced clinical neuroradiologist (with 10 years’ experience), who assessed multiple modalities of neuroimaging data (T1-weighted, SWI, FLAIR) for all subjects in random sequence. The neuroradiologist was blind to clinical information and group membership (patient or control).

### Preprocessing of Resting-State fMRI Data

Data processing was performed using SPM12 (Statistical Parametric Mapping, University College of London, London, UK) on MATLAB platform (R2013a; MathWorks, Natick, MA, USA). Image preprocessing steps included anatomical segmentation, normalization to Montreal Neurological Institute (MNI) space, spatial smoothing (full-width at half-maximum, FWHM = 6 mm), band-pass filtering (0.01–0.1 Hz), and regressing out signal contributions from head motion, white matter, and cerebrospinal fluid (Thompson et al., 2016). We adopted a seed-based method to extract the DMN. Regions of interest (ROI) in the DMN were defined based on a previous task fMRI study (Duan et al., 2012). In our study, we selected only the PCC (MNI coordinates: 0, −52, 27) as the seed for brain network connectivity analysis. Next, for each subject, the time series of the mean BOLD signal of a 10-mm radius sphere centered at the peak coordinate of ROI was extracted to calculate the functional connectivity with each voxel of the whole brain. Prior to statistical analysis, the functional connectivity maps were transformed into *z*-values using Fisher transformation to improve normality. We then performed conjunction analyses to identify brain areas that were positively correlated with the PCC seed. A family-wise error (FWE) method with a threshold of *P* < 0.05 was set for multiple comparisons. A voxel-wise one-way analysis of variance (ANOVA) was used to test for network functional connectivity differences (*P* < 0.05, FWE correction) across three groups with gender as a nuisance covariate within DNM mask. We then used *post hoc* analyses to test for network functional connectivity differences (*P* < 0.05, FWE correction) across each of the two groups.

### Statistical Analysis

All statistical analyses were performed using SPSS (version 19, IBM Corp, New York, NY, USA) and Prism (Version 7, GraphPad Software, San Diego, CA, USA). The Shapiro–Wilk W test was used to test for normality distribution in all continuous variables. The independent two-sample *t*-test and the Mann–Whitney test were used to compare group differences based on data normality. Chi-square analyses were applied to assess categorical variables. Multivariate linear regression analysis was used to determine the association between the levels of α-syn and neuropsychological testing. The levels of α-syn, age, gender, and education years were entered into the model as independent variables, with results of cognitive tests in the mTBI patients as dependent variables.

## Results

### Participant Characteristics

Fifty-two patients with mTBI (27 males, age of 34.48 ± 13.32 years, education level of 9.31 ± 4.36 years) and 47 matched healthy controls (22 males, age of 35.43 ± 12.01 years, education level of 11.39 ± 5.66 years) were recruited for this study. No significant differences existed between the mTBI patients and healthy controls regarding age, education level, and gender (*p* > 0.05). The detailed demographic data and clinical characteristics of the participants are summarized in Table 1. Upon arrival at the emergency department, all patients with mTBI had an initial GCS of 15. The causes of injury included motor vehicle accidents (58%), assaults (25%), and falls (17%). All the mTBI patients had negative computed tomography findings.

**Table 1:** Summary of demographic characteristic and neuropsychological test scores between patients and controls at acute phase.

	mTBI patients	Controls	*P*-value
**Demographic**			
Age	34.5 ± 13.3 (14–63)	35.4 ± 12.0 (14–60)	0.462
Gender (M/F)	27/25	22/25	0.258
Education	9.3 ± 4.4	11.4 ± 5.7	0.062
α-syn	536.9 ± 152.4	517.2 ± 86.3	0.470
**Neuropsychological test**			
TMT A	60.5 ± 43.5	46.3 ± 33.2	0.073
FDS	8.1 ± 1.6	8.5 ± 1.5	<0.001*
BDS	4.1 ± 1.5	4.6 ± 1.9	0.458
LF	17.3 ± 5.4	18.9 ± 5.9	0.280
DSC	37.9 ± 16.2	47.2 ± 16.9	0.007*
**Self-report measures**			
PCS	10.6 ± 7.1	2.1 ± 2.6	<0.001*
PCL-C	25.0 ± 6.3	17.0 ± 0.0	<0.001*
FSS	10.4 ± 5.7	9.0 ± 0.0	0.054
BDI	4.7 ± 3.7	0.1 ± 0.2	<0.001*
ISI	6.9 ± 6.2	1.9 ± 3.1	<0.001*
**mTBI severity (N%)**			
GCS = 15	52 (100%)		
GCS = 13, 14	0 (0%)		
**Causes for mild TBI (N%)**			
Motor vehicle accident	30 (58%)		
Assaults	13 (25%)		
Fall	9 (17%)		

TMT A, Trail-Making Test Part A; FDS, Forward Digit Span Task; BDS, Backward Digit Span Task; LF, Language Fluency; DSC, Digit Symbol Coding; PCS, Postconcussive Symptoms Scale; PCL-C, Post-Traumatic Stress Disorder Checklist Civilian; FSS, Fatigue Severity Scale; BDI, Beck Depression Inventory; ISI, Insomnia Severity Index; GCS, Glasgow Coma Scale. *p < 0.05.

We divided the mTBI patients into two groups according to the split criteria of whether the α-syn levels were above or below the mean plus one standard deviation α-syn level in the healthy controls. Therefore, patients with α-syn levels lower than 536.93 pg/mL were categorized into the Patient-A group, and those with α-syn levels higher than 536.93 pg/mL were divided into the Patient-B group. Patient-A consisted of 25 patients (12 male, age of 33.92 ± 13.67 years, education level of 9.40 ± 4.15 years), and Patient-B consisted of 27 patients (15 males, age of 35.00 ± 13.24 years, education level of 9.22 ± 4.62 years). No significant differences occurred between Patient-A and B in terms of age, education level, and gender (*p* > 0.05). The detailed demographic data and clinical characteristics of the patients per group were summarized in Table 2.

**Table 2:** Summary of demographic characteristic and neuropsychological test scores between patients and controls at acute phase.

	Patient-A	Patient-B	*P*-value
**Demographic**			
Age	33.9 ± 13.7 (17–60)	35.0 ± 13.2 (14–62)	0.479
Gender (M/F)	12/13	15/12	0.586
Education	9.4 ± 4.2	9.2 ± 4.6	0.941
α-syn	401.3 ± 66.3	662.5 ± 96.1	<0.001*
**Neuropsychological test**			
TMT A	62.0 ± 49.4	46.3 ± 33.2	0.679
FDS	8.2 ± 1.6	8.5 ± 1.5	0.718
BDS	4.2 ± 1.5	4.6 ± 1.9	0.652
LF	16.8 ± 5.9	18.9 ± 5.9	0.377
DSC	38.0 ± 14.5	47.2 ± 16.9	0.762
**Self-report measures**			
PCS	13.4 ± 8.3	2.1 ± 2.6	0.012*
PCL-C	25.6 ± 7.9	17.0 ± 0.0	0.612
FSS	12.0 ± 8.0	9.0 ± 0.0	0.026*
BDI	5.6 ± 4.2	0.1 ± 0.2	0.096
ISI	8.0 ± 6.7	1.9 ± 3.1	0.161
**mTBI severity (N%)**			
GCS = 15	25 (100%)	27 (100%)	
GCS = 13, 14	0 (0%)	0 (0%)	
**Causes for mild TBI (N%)**			
Motor vehicle accident	14 (56%)	16 (59%)	
Assaults	4 (16%)	9 (33%)	
Fall	7 (28%)	2 (8%)	

TMT A, Trail-Making Test Part A; FDS, Forward Digit Span Task; BDS, Backward Digit Span Task; LF, Language Fluency; DSC, Digit Symbol Coding; PCS, Postconcussive Symptoms Scale; PCL-C, Post-Traumatic Stress Disorder Checklist Civilian; FSS, Fatigue Severity Scale; BDI, Beck Depression Inventory; ISI, Insomnia Severity Index; GCS, Glasgow Coma Scale. *p < 0.05.

### Neuropsychological Measures

Compared to the controls, the mTBI patients exhibited lower performance in terms of information processing speed while performing DSC task (*p* = 0.007), digit span forward (*p* < 0.001), and verbal fluency tasks rated by language fluency (*p* = 0.013). Moreover, the patients presented more complaints in the Post-Traumatic Stress Disorder Checklist (Civilian Version) compared to the controls (*p* < 0.001) and showed significant difference in the Post-Concussion Symptom Scale (*p* < 0.001), BDI (*p* < 0.001), and ISI (*p* < 0.001; Table 1).

### Relationship of Serum α-Syn With Neuropsychological Characteristics

**Figure 1:**
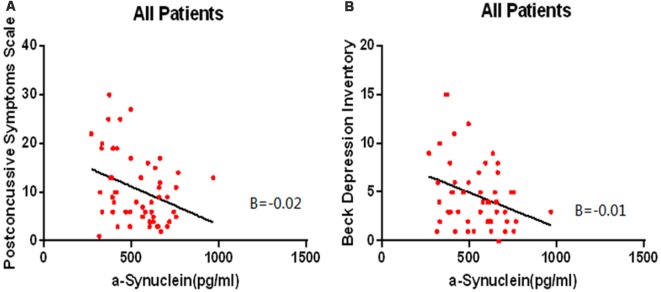
The lower levels of α-syn are associated with severer outcomes from the Post-Concussion Symptom Scale (*β* = –0.333, *p* = 0.013, overall model: *F*_(3,48)_ = 3.259, *p* = 0.019, adjusted *R*^2^ = 0.217, **A**) and severer depression according to the Beck Depression Inventory (BDI; *β* = –0.311, *p* = 0.022, overall model: *F*_(3,48)_ = 2.840, *p* = 0.034, adjusted *R*^2^ = 0.195, **B**) in the mTBI patients.

Multiple linear regression analysis showed that lower levels of α-syn in the mTBI patients are associated with severer outcomes from the Post-Concussion Symptom Scale (*β* = –0.333, *p* = 0.013, overall model: *F*_(3,48)_ = 3.259, *p* = 0.019, adjusted *R*^2^ = 0.217, Figure 1A) and severer depression according to the BDI (*β* = –0.311, *p* = 0.022, overall model: *F*_(3,48)_ = 2.840, *p* = 0.034, adjusted *R*^2^ = 0.195, Figure 1B).

**Figure 2:**
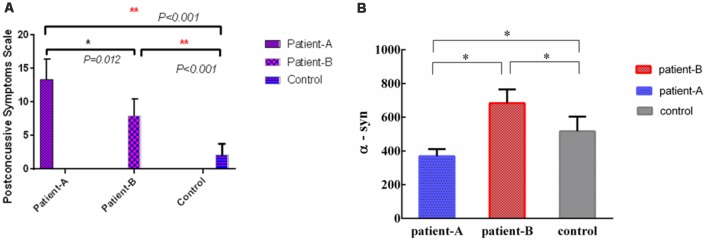
The levels of serum α-syn of Patient-A was significantly different with Patient-B (*p* = 0.012) and controls (*p* < 0.001), meanwhile, the levels of serum α-syn of Patient-B was significantly different with controls (*p* < 0.001; **A**). The levels of serum α-syn in Patient-A were significantly different compared with Patient-B (*p* < 0.001), the levels of serum α-syn in controls were also significantly different compare with Patient-A (*p* < 0.001) and Patient-B (*p* < 0.001; **B**). *p < 0.05, **p < 0.001.

The α-syn levels in Patient-A were significantly different compared with Patient-B (*p* < 0.001), and the α-syn levels in controls were significantly different compared to both Patient-A (*p* < 0.001) and B (*p* < 0.001; Figure 2B). The significant differences persisted after multiple corrections.

The α-syn in serum of Patient-A patients was significantly lower than those of Patient-B patients (*p* = 0.012) and controls (*p* < 0.001). Meanwhile, the α-syn in the serum of Patient-B patients was significantly higher than that of controls (*p* < 0.001; Figure 2A).

### Default Mode Network Results

**Figure 3:**
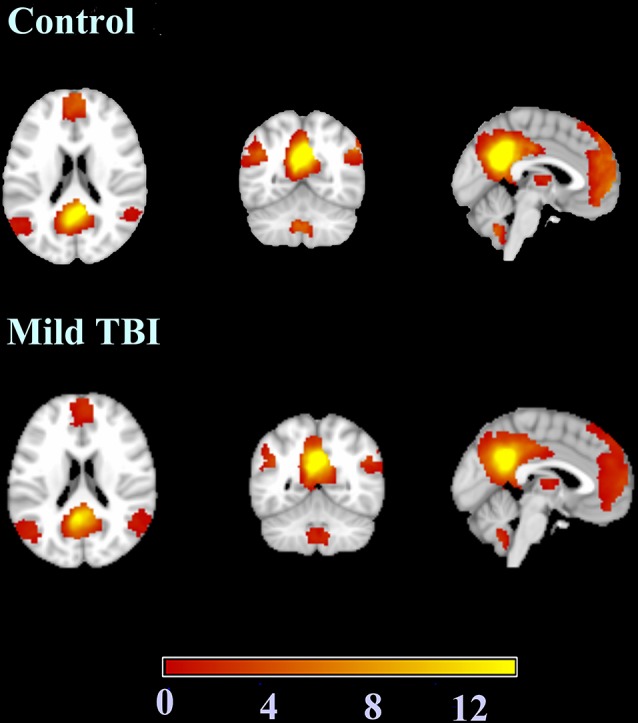
The results of the one-sample *t*-test used to identify brain regions within the default mode network (DMN) mask with functional connectivity differences.

**Figure 4:**
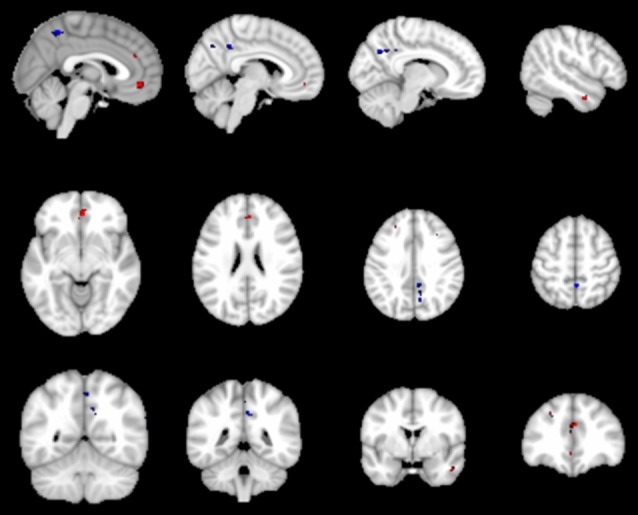
The red represent regions of increased rs-FC in the Patient-B compared with Patient-A and the blue represent regions of reduced rs-FC. Cluster extent threshold of *p*-value of 0.05 using a family-wise error (FWE) correction for multiple comparisons.

The results of the one-sample *t*-test used to identify brain regions within the DMN mask with functional connectivity differences (*p* < 0.05, FWE corrected) in the participants are shown in Figure 3. We also compared the changes in the DMN between the two groups, and Patient-B presented a prominently higher functional connectivity in the left anterior cingulate cortex and ventral MPFC (*p* < 0.05, FWE corrected), and a significantly lower functional connectivity in the precuneus (*p* < 0.05, FWE corrected) compared with Patient-A (Figure 4). There was also no significant correlation between functional connectivity and serum α-syn.

## Discussion

In this study, we explored α-syn as candidate biomarker and combined it with brain imaging data for both diagnosis and prognosis of patients suffering from mTBI. Compared with the healthy control group, the mTBI patients had lower scores for FDS and DSC, and higher scores for PCS, PCL-C, BDI and ISI. These results were consistent with neuropsychiatric symptoms of the mTBI patients: persistent headaches, difficulty thinking, memory deficits, inattention, emotional instability, and brief frustration after injury. We found that patients with lower α-syn levels exhibited severe post-concussion and depressive symptoms. Moreover, patients with higher α-syn levels, compared to those with lower α-syn levels, showed significantly increased functional connectivity in the left anterior cingulate cortex and ventral MPFC, and a significantly decreased connectivity in the precuneus. To the best of our knowledge, this is the first study to examine α-syn level and its association with the DMN during the early acute phase of mTBI, and explore the relationship between α-syn levels and cognitive function, which are major determinants of poor prognosis in mTBI.

The presynaptic terminals have plenty of neuronal proteins, one being α-syn (Vekrellis et al., 2011), which is the main component of Lewy bodies. The accumulation of α-syn aggregates is also found in Lewy body dementia and multiple system atrophy (Spillantini et al., 1998). Although several studies have examined the levels of α-syn in cerebrospinal fluid (Mollenhauer et al., 2011, 2013) or serum (Lin et al., 2017) of the mTBI patients, their results were inconsistent. Subsequently, reduction of costs, development of noninvasive techniques, and methodological advances have enabled more intensive and deep serum studies (Lin et al., 2017; Singh et al., 2018). Prior studies have investigated the relationship between TBI and the risk of developing PD (Kenborg et al., 2015; Perry et al., 2016; Taylor et al., 2016). It has been reported that mTBI is associated with a 56% increased risk of developing PD, even after adjusting for demographics and medical comorbidities among military veterans (Gardner et al., 2018). In this study, we found that patients with lower α-syn in serum exhibited severer post-concussion and depressive symptoms during the early acute phase of mTBI. Although past research on animals found that overexpression of α-syn may constitute a pathological link between TBI and development of pathologies similar to PD (Stewart et al., 2014), that study adopted male rats, whose brain function and structure notably differ from those of humans. In addition, the subjects of our study were recruited within the 1st week after suffering mTBI. We hypothesized that during the acute phase of mTBI, elevated α-syn is a product of compensatory mechanisms, which means lower α-syn patients may be unable to compensate these levels after mTBI, leading to severer post-concussion and depressive symptoms. Schilbach et al. (2008) suggested that decreased levels of cerebrospinal fluid α-syn in early stage PD may be the result of cellular compensatory mechanisms for pathological soluble α-syn, thus obtaining lower levels of α-syn compared with controls (Schilbach et al., 2008).

The DMN is a task-negative network involving self-reference processes, such as introspection and experiential memory (Schilbach et al., 2008; Fingelkurts and Fingelkurts, 2011). The essential nodes of the DMN include the PCC, bilateral angular gyri, precuneus, ventro-MPFC, and dorsal-MPFC. The functional connectivity within the DMN has been related to performance in higher cognitive functions such as attention and memory (Hampson et al., 2006; Buckner et al., 2008; Leech et al., 2011); meanwhile, cognitive dysfunctions are the most commonly reported consequences of mTBI (van der Naalt et al., 1999; Smith-Seemiller et al., 2003). In addition, fMRI-based studies have illustrated the DMN dysfunction after mTBI (Palacios et al., 2013; Nathan et al., 2015), and its relation to several diseases such as Alzheimer’s disease, attention-deficit/hyperactivity disorder, and schizophrenia (Whitfield-Gabrieli et al., 2009; Koch et al., 2012).

Few studies have explored the causes of connectivity dysfunction within the DMN. Our results showed decreased functional connectivity between the anterior cingulate cortex and ventro-MPFC in patients with lower α-syn in serum compared to those with higher α-syn in serum. In addition, patients with lower levels of α-syn suffered significantly severer post-concussive and depressive symptoms. The brain regions involved in the DMN are related to high-level cognitive functions and decision-making. For instance, the precuneus is involved in learning and memory (Price, 2000). The anterior cingulate cortex has been associated with the anxiety disorder, post-traumatic stress disorder (Kennis et al., 2015), while MPFC has been found to participate in self-relevance, rapid error identification, and social functions. Moreover, changes in functional connectivity may be due to spontaneous seizures of neuronal ensembles, changes in blood flow, oxidative metabolism, or combinations of these factors (Fox and Raichle, 2007). Furthermore, α-syn is a presynaptic protein activated by various processes such as phosphorylation and contributes to the synaptic vesicle cycle (Acosta et al., 2015). These findings suggest that α-syn in serum can modulate the connectivity of the DMN in patients with mTBI.

Several limitations of our study remain to be addressed. First, we only explored changes in the DMN functional connectivity within the 1st week post-injury. However, the considered post-injury period should be longer to accurately observe the functional connectivity evolution, and longitudinal analyses need to be conducted using follow-up data. Second, while α-syn concentration in serum changed significantly during the acute phase, the 1-week period post-injury provided limited information for observing the dynamic changes of α-syn in the acute phase. Third, we only considered resting-state fMRI, and the results did not exclude the possibility of structural changes, which can be measured by diffusion tensor imaging. Future research must explore the relationship between structural and functional network defects and their clinical significance.

## Conclusion

We found that mTBI may involve a chronic, progressive, neurodegenerative process, and may be closely associated with the occurrence of PD. As biomarker of PD, α-syn in serum also exhibits significant changes in the mTBI patients. Specifically, patients with higher levels of α-syn in serum exhibited increased functional connectivity in the left anterior cingulate cortex and ventro-MPFC and decreased functional connectivity in the precuneus. Therefore, α-syn in serum may be considered as one of the modulations of the DMN connectivity in patients with mTBI. These results suggest the plausibility of using the α-syn in serum as objective biomarker for the prognosis of mTBI and early intervention to prevent adverse sequels in patients with this type of injury.

## Ethics Statement

All the subjects gave written, informed consent in person approved by a local institutional review board and conducted in accordance with the Declaration of Helsinki.

## Author Contributions

LY, ZY, and GB contributed to the conception of the study. LY contributed significantly to analysis and manuscript preparation. LY, DZ, PZ, and MS performed the data analyses and wrote the manuscript. CG, BY, JZ, and FW helped perform the analysis with constructive discussions.

## Conflict of Interest Statement

The authors declare that the research was conducted in the absence of any commercial or financial relationships that could be construed as a potential conflict of interest.
